# Adult Maltese Women’s Understanding of How Childhood Domestic Violence Has Impacted Their Relationships with Their Parents and Siblings: A Grounded Theory Study

**DOI:** 10.3390/bs14040333

**Published:** 2024-04-16

**Authors:** Clarissa Sammut-Scerri, Arlene Vetere

**Affiliations:** 1 Department of Child and Family Studies, Faculty for Social Wellbeing, University of Malta, MSD 2080 Msida, Malta; 2 Faculty of Social Studies, Vid Specialised University, P.O. Box 184, Vinderen, 0319 Oslo, Norway; drarlenevetere@hotmail.com

**Keywords:** parents, siblings, domestic violence, adult women, qualitative research, grounded theory, parent-child relationships, recollections, complexity, love

## Abstract

Most of the literature that has looked at children’s relationships with their parents in the domestic violence context has focused solely on the children’s relationship with one parent or is studied from the perspective of one parent, usually the mother. Sibling relationships in the same context are also under-studied. This paper explores in more detail the complexity of children’s relationships with their mothers, fathers, and siblings over time from the perspective of adult women and survivors of childhood domestic violence. Methods: A grounded theory methodology was used to analyse the interviews with 15 women aged twenty to forty-three years of age living in Malta. Results: the analysis showed that the domestic violence context remains significant in these important relationships for these women. The relationship with the father remains strongly influenced by the dynamics of fear, love, and retaliation, with cycles of cut-off and connection from the adult daughter’s end. The relationship with the mother is complicated—feelings of love that are seen as having been limited and complicated by betrayal if there was abuse from the mother. Similarly, for the siblings, the roles of the early family of origin remain persistent and significant. However, in some of these relationships, there has been transformation, reconciliation, and forgiveness. The article offers implications for therapeutic practice for dealing with the complexity of these relationships and ideas for future research.

## 1. Introduction

This paper presents the results of a qualitative research study exploring adult women’s recollections of their relationships with their parents and siblings while living in a family where there was domestic violence as children. It also presents their reflections on these relationships across time and from their perspective as adults. The results are part of a larger study that was guided by a constructivist grounded theory methodology [1] that investigated adult women’s understanding of their childhood experiences of domestic violence using a systemic relational perspective [2].

In the absence of an agreed-upon definition of what constitutes domestic violence or domestic abuse [3], and in the recognition that coercive control is central to the perpetration of domestic violence [4,5], domestic violence will be defined as follows: “Any incident or pattern of incidents of controlling, coercive or threatening behaviour, violence or abuse between those aged 16 or over who are or have been intimate partners or family members regardless of gender or sexuality. This can encompass but is not limited to, the following types of abuse: psychological, physical, sexual, financial, and emotional. Controlling behaviour is defined as a range of acts designed to make a person subordinate and/or dependent by isolating them from sources of support, exploiting their resources and capacities for personal gain, depriving them of the means needed for independence, resistance and escape and regulating their everyday behaviour. Coercive behaviour is an act or a pattern of acts of assault, threats, humiliation and intimidation or other abuse that is used to harm, punish, or frighten their victim” [6].

The UK Domestic Abuse Act [7], from which the definition of domestic abuse was taken, also specifically recognises the devastating consequences that domestic violence can have on children by referring to children as victims of domestic abuse, t when they are related to the adult victim or the suspect who perpetrates the domestic violence [7].

We have opted for this definition because it clearly describes the kind of behaviour that is considered abusive; it acknowledges that domestic violence can happen in various forms, that it is not limited by gender or sexuality, that it can take place between intimate partners and family members and specifically acknowledges children as victims of domestic violence. The latest European prevalence study available at the time of writing shows that out of all women who have a partner (current or previous), 22% have experienced physical and/or sexual violence by a partner during their lifetime since the age of 15 years [8]. This percentage is slightly lower than the latest Maltese prevalence rate, where 26.0% of those women who had or have an intimate partner have experienced at least one episode of intimate partner violence during their lifetime since the age of 15 years [9]. As for the prevalence rates of children witnessing domestic violence, Maltese participants in an online survey with a representative sample of 18 to 24 years old in Malta have indicated that 46% of the sample have witnessed their parents’ intimate partner violence, that is, physical abuse or psychological abuse or property damage during their childhood [10].

In the past 40 years, quantitative research on children and domestic abuse has clearly mapped the far-reaching harmful and negative effects it has on their physical, emotional, and psychological development, their academic competence and even their health [11]. All this comes at a considerable cost to the children and their families living in domestic violence (DV), as well as to society at large. Nevertheless, it has to be said that not all children experience these negative sequelae, and some children fare well despite the exposure to violence [12,13]. For this reason, we argue that children need to be seen in the totality of their experiences and not just as victims and not merely as “witnesses” but as actually living the domestic violence experience [5,14].

In the last 20 years, qualitative research has started illuminating what it is like for children to live in a family where there is domestic violence. Children often need to contend with the unpredictability of violence at home [15,16,17]. There might be days when they might see their father hit their mother and their siblings, and they might often be maltreated themselves. But there might also be good days at home, with no violence. In the middle of all this, there are the children’s relationships with their frightened parents (because of the domestic violence they experience), who in turn may be frightening to the children because of the abuse that they put them through [18].

Most of the literature that has looked at children’s relationships with their parents has focused solely on children’s relationships with one parent, and even here, there is considerable variation in the children’s experiences [19]. There is significantly more literature on the relationship between mothers and their children than on fathers-to-child relationships [20]. One explanation could be perhaps because mothers are still socially constructed as primarily responsible for their children’s psychological well-being and for their increased resilience [5,13]. In addition, fathers may still be mainly constructed as the “oppressive other” [3] (p. 121). Research studies portray fathers as lacking in warmth and responsiveness, disengaged and abusive to children and older young adult children [21]. However, some children acknowledge a desire to be close to their father, holding simultaneously mixed, ambivalent, and contradictory views of him [4,22,23,24,25,26]. Perhaps this is children’s way of attempting to maintain a positive relationship with him [27]. Children are also capable of a sophisticated analysis of their father—seeing him as someone who is capable of being “nice” to others, “nice to them”, but also bad to their mother, depending on who he is with and on the circumstances [28].

The literature on the relationship with the mother is also varied, and most studies are from the perspective of the mothers rather than from the children’s perspective [20]. In the few studies that have explored the children’s perspectives, some children feel that their mother, as the non-offending parent, is their protector and that they are emotionally close to her [22]. Other children acknowledged the difficult relationship that they had with their mother—in that she had not been available to look after them, and/or her needs took priority over those of the children when they felt that they had to protect her from the violence. Other children saw their mothers as weak or crazy and to blame, highlighting the strain that the domestic violence context puts on the children’s relationship with their mothers [29]. Very few studies look at these relationships in the context of each other. One exception can be found in Katz’s (2019) study, which briefly looked at five interlinked factors that influenced the levels of closeness, distance, and strain in the mother-child relationships from the perspective of the mothers and the children [20]. These factors include the father’s behaviour towards the children, the father’s use of domestic violence, the father’s undermining of the mother-child relationship, the mother’s ability to emotionally connect to her children and the children’s views of the mother and the father.

Most studies about children’s experiences of domestic violence focus on a single child and overlook sibling relationships [5,30,31]. This is surprising given that sibling relationships are usually one of the most long-standing relationships in one’s lifetime [32]. Sibling relationships, especially in the context of domestic violence, are important for the children’s psychological adjustment and may offer protection to their psychological development or, conversely, increase the risk factors [30,33,34]. Sibling relations are more often studied in the context of sibling violence or in the context of abuse and neglect [35], separate from the context of domestic violence. Reactions to sibling aggression vary widely between being viewed as harmless and inconsequential [36,37,38] or of serious concern for parents and clinicians [39].

The few studies that have explored siblings’ relationships in the context of domestic violence describe the strong, caring roles that children take towards their mother and their siblings [5,17] both in terms of their relationships but also as a function of living with and coping with violence [5]. More recent work has also addressed the higher risk of sibling aggression in children living in domestic violence [40,41], with the study by Piotrowski, Tachie and Cameranesi (2021) investigating sibling aggression from a multi-informant perspective: the mothers’ [42], the siblings themselves and observers. It is also important to note that half of the sample of this latter study displayed no aggression at all, again highlighting the importance for practitioners holding both a trauma and a resilience perspective [5,43].

The aim of this paper is to explore in more detail the complexity of the children’s relationships with their mothers, fathers, and siblings over time, with the added benefit of adult reflection that can enlighten possible ways to help children with experiences of domestic violence.

### 1.1. Theoretical Ideas That Guided the Development of the Research Question and the Data Analysis

#### 1.1.1. Cognitive-Contextual Framework

Grych’s and Fincham’s framework [44] proposes that when children witness an interpersonal conflict, they try to understand how the perceived threat may affect them, why it is happening and what they can do as a result [45]. This model also takes into account contextual factors that may affect the children’s perception of threat, such as the way that the conflict is expressed, whether disagreements concern a child-related issue, the impact of the parent-child relationship and also wider contexts such as exposure to violence in the community and the impact of culture. When the framework was studied in families where there was violence, studies showed that children’s appraisals guide both their immediate response to domestic violence as well as shape their beliefs and expectations about aggression and about close relationships in general [45]. One belief is the acceptability of aggression or the extent to which violence is seen as justifiable. Children might witness incidents where the person who is aggressive gets his or her way, or the children might perceive the parent who is abused as somehow responsible for being victimised or see the use of aggression as a form of self-defence. Depending on these beliefs and how children understand violent interactions might explain why some children may perpetuate aggressive behaviours in relationships while others might become anxious or depressed and may avoid close relationships. However, the mechanism of why some children follow one path and not another is still unclear.

#### 1.1.2. Emotional Security Theory

Consistent with attachment theory [46,47,48,49], which highlights the importance of the parent–child relationship, this theory [50] explains that families provide children with a sense of protection, safety and security [45,51]. However, when parents’ interpersonal relationship is conflictual or when parents are sources of fear and threat, as might happen in families where there is domestic violence, children lose their sense of emotional security, and they may become fearfully aroused. In this context, children may be motivated to try and regulate their parents’ behaviour either by misbehaving or by directly intervening to stop the marital conflict. This is similar to the concept of triangulation in the family system [52,53] This theory also looks at children’s past experiences with marital conflict as internal representations, which then play an important role in their adjustment to conflict, including how they assess conflict and threat, why it is occurring, who is responsible and whether they have skills for coping [54].

#### 1.1.3. Coercive Family Processes

According to coercion theory [55], parents and children can be “stuck” in predictable, negative interaction patterns where both engage in aversive behaviour. A typical coercive cycle would begin when a parent reprimands a child for misbehaving, which would, in turn, result in the child misbehaving further. If the parent then disengages to stop the child’s aversive behaviour, then both participants have been shaped by this response. The parent has been shaped to give up when the child misbehaves, and the child has been shaped to misbehave again when the parent exerts discipline. These parent-child interactions become more problematic as they increasingly become more difficult to manage, possibly leading to the escalation of aggressive behaviour over time. There is also a tendency for parents who are abusive to be inconsistent in responding to child non-compliance, but when they do respond, they are more likely to use power and coercive strategies [56].

#### 1.1.4. Family Systems Theory

One of the key ideas of the systemic framework is the emphasis on interpersonal relationships and relational processes as opposed to an intrapsychic focus [57]. One positive aspect of an interpersonal perspective is that family members are not seen as determined by their past experiences. This is consistent with a resilient framework, which also guided this study [58]. In addition, another important concept is that of looking at triangles or three-person interaction as a unit of analysis [52], implying that what happens between two people in a family can have an important influence on a third person and their relationship, and vice-versa. This is relevant in this study as participants talk about their understanding of their parents’ relationships and those with their siblings. Finally, with the influence of constructivism and social constructionism, the impact of cultural and societally shared beliefs also became increasingly important to understand. In this study, importance is given to participants’ gendered beliefs, which might have developed because of their experiences and as a result of their life experience in a Maltese culture. Thirty years ago or so, when the participants were still children, Maltese society strongly upheld Catholic values [59]: problems in marriage and filing for separation were seen as undesirable and shameful, even in the contexts of domestic violence, and children were expected to respect and honour their parents, above everything else. Thus, systems theory allows for the formulation of different processes on the level of the individual, the family, and the social level. However, this approach has also been criticised as overlooking the role of emotions [52]. In this sense, it is useful to look at an attachment perspective with a focus on social and emotional development in a relational context.

#### 1.1.5. Attachment Theory

This section will focus on key contributions from Bowlby’s work [46,47,48,49], Main’s [60], Ainsworth’s [61] and Crittenden’s Dynamic Maturational Model (DMM) of attachment [62,63]. According to Bowlby, when significant others are available in times of need and provide sensitive and attuned responses to children, especially in situations of perceived danger, children develop a sense of attachment security. Conversely, when caregivers are unpredictable or not available, as might happen with domestic violence, the children’s distress is not relieved, and they do not attain attachment security nor learn how to manage their emotions and calm themselves down [64]. Critics of Bowlby’s theory have sometimes argued that such a framework might be too deterministic and rather pathologising. Instead, the DMM model appreciates that attachment is not fixed early in life and that it becomes increasingly complex with maturation and experience [62,63]. One of the central ideas of the DMM model is that the key motivation for attachment behaviour is danger rather than safety. This means that what Main, Ainsworth and colleagues [60,61] would term disorganised attachment patterns, the DMM model would understand these as self-protective strategies that will change when it is safe for them to behave in alternative ways. This approach is non-pathologizing and can be used to understand complex behaviour in contexts of danger and threat.

#### 1.1.6. Trauma Theory

Exposure to chronic family violence has been linked to post-traumatic stress order (PTSD) symptoms [65,66], helping practitioners understand some aspects of their client’s experiences, especially if children have been exposed to death, threatened death or actual or threatened serious injury or sexual violence—all events that are likely to take place in a family where there is domestic violence. However, limiting trauma to exposure to actual threat or threatened death, serious injury or sexual violence does not say anything about chronic, intergenerational abuse that people might face in domestic violence contexts [66], where children might be reacting to continuous events, or they may be reacting to triggers. In addition, children might also experience events which, although may not be life-threatening, be experienced as equally upsetting, such as experiencing verbal abuse, being coerced, or being humiliated. The diagnosis of complex trauma [67] might better explain child and adult survivors’ difficulties with anxiety and depression as well as problems with affect regulation, managing relationships, substance abuse, and self-harming behaviour.

It is useful to keep in mind that although such theoretical frameworks may help widen clinicians’ understanding of the cumulative trauma responses in children, not all children who experience domestic violence fare poorly when compared to those children who do not witness domestic violence. It is also important to look at resilience and the notion of protective factors as part of their experiences of witnessing domestic violence [68].

#### 1.1.7. A Resilience Framework

Resilience is here taken to refer to a “dynamic process encompassing positive adaptation within the context of significant adversity” ([58] p. 543). This broad definition highlights the dynamic perspective of resilience, which means that people can be resilient in some adverse contexts but not in others. In addition, resilience is not a trait or quality of an individual but rather a process of positive adaptation within an adverse context [58] involving a complex interplay between genetic and environmental influences [69,70]. Nurturing relationships such as with one ‘s parents or other significant others can be important protective processes that can give rise to resilient coping [71]. A warm, safe, and sensitive relationship context can support the development of affect regulation, a view of others as helpful and trustworthy, and the capacity to deal with complex information.

## 2. Methodology

As stated above, this paper is based on a larger study [2] that was guided by a constructivist grounded theory methodology [1]. This methodology consists of a set of inductive steps that enable researchers to focus on their concurrent data collection and data analysis through constant comparison analysis to build inductive middle-range theories through successive levels of data analysis at more abstract and conceptual levels [72,73]. Constructivist grounded theory acknowledges that the data are jointly constructed between researchers and participants and gives space for the researcher to see beyond their point of view and privilege [74]. The construction of data in the form of in-depth, semi-structured interviews also gives participants the opportunity to discuss topics and issues from their point of view. From the interviews, it was apparent that recollections and reflections about their relationships with their parents and siblings were a significant part of these women’s understanding of what it was like for them to live in a family with domestic violence.

### 2.1. Sample

The participants consisted of 15 persons who identified as women between the ages of 20 and 43 years, with an average age of 28.5 years. They were well-functioning women in paid employment and had significant relationships in their lives. Three of the participants—Anita, Geraldine, and Carmen (all women’s names in the articles are pseudonyms) had resided in a children’s out-of-home facility for part of their childhood. None of the women have been in a shelter for women with domestic violence issues. At the time of the interviews, none had a history of substance abuse.

The following table (Table 1) shows the participants’ demographic details and conveys a picture of their varied backgrounds.

Table 2 (below) helps the reader understand the context of abuse that the participants experienced both in witnessing their parents’ intimate partner violence and the abuse they directly experienced as children.

Almost all the participants (13 out of 15 women) lived in a family where physical and emotional abuse was perpetrated by the father on the mother. Two participants—Farrah and Tori, perceived their parents as mostly engaging in the emotional abuse of each other. In addition, all women except Tori were physically and emotionally abused by their father, and six participants were also physically and emotionally abused by their mother. Seana was also maltreated by her siblings, and Marika admitted to being aggressive with her younger sibling. This table highlights the different forms of domestic violence that often occur in families and how child abuse is often found to be significantly associated with intimate partner violence [75].

### 2.2. Recruitment

Prior to starting the data collection, approval was obtained from the relevant university research ethics review committees of the first author and the relevant social welfare agency research ethics committee. Given the sensitivity of the topic and the potential for the data collection process to re-evoke unprocessed trauma memories, participants were recruited through health and social care professional networks. Out of duty of care and professional collegiality, these professionals also offered to support the participants should they feel uncomfortable after the interviews due to the interventive nature of the questions [76]. As the data collection and analysis progressed, it was important that the participants had the opportunity to discuss and process any trauma memories that might have been recalled in the interviews and by the interviewing process further.

### 2.3. Data Collection and Analysis

The interviews were guided by a semi-structured interview guide, based on questions from the Adult Attachment Interview [77] and with reflexive and circular questions [78,79] but with flexibility so that the women could raise the issues that they saw as important and relevant. The AAI was not used to code the participants’ transcripts according to their attachment strategies, but the questions were used to help participants retrospectively reflect on their childhood experiences of domestic violence and their understanding of the impact these experiences have had on their relationships and their individual development.

All the interviews, except one, were held in Maltese. One interview was held in English as the participant was not Maltese, but she was married to a Maltese. Each interview was transcribed verbatim and translated into English in order for both authors to follow and for the analytic audit trail. The accuracy of the translation was checked with backward and forward translation [80]. In line with the grounded theory methodology, each interview was coded line by line, and initial codes were noted and discussed with the co-author. As the interviews progressed, a constant comparative method was used to compare similarities and differences between existing data and emergent data to further refine codes. The data were further summarised and coded into focused codes. The authors noted down their thoughts, different interpretations, and links to theoretical concepts in the form of memos. Memos were also used to start linking categories and sub-categories [1] and to hypothesise relationships between different categories. As the authors engaged in further analysis, they felt confident in proposing the process of “Living with contradictions, double binds and dilemmas of love and abuse” as a core category of the study. The relationships between different categories were hypothesised as a model illustrating the women’s understanding of the links that they made between their childhood experiences and their adult development and relationships [2].

### 2.4. Credibility Checks

One way of ensuring the trustworthiness of the data was the emphasis on reflexivity, which helped the authors examine their biases and assumptions. Two reflexivity interviews were held with the first author during the start of the research process and in the middle of the process. One of the interesting themes that emerged was how unexpected and shocking it was to hear the women talk about love and connection with the father after having listened to horrific stories of abuse and cruelty. It was hard to keep these two contradictory notions together. It was hypothesised that this was what the participants must have felt and still feel in relation to their experiences. As another form of credibility check, two discussion groups were conducted with health and social care professionals working in the domestic violence field. Their feedback provided support for the credibility of the analysis as the findings made sense to them and were useful to their practice. Following the completion of the analysis, member checks were also conducted with three of the participants who were interested in the results and who also gave feedback that the hypothesised model represented their experiences well.

## 3. Findings

Before presenting the participants’ reflections on their relationships with their parents and siblings, it is important for the reader to understand the context in which these relationships were situated. The participants also stated that it was important for the interviewer (first author) to understand the kind of violent experiences they went through as children. The findings are also interpreted from the perspective of previous research studies.

### 3.1. The Context for Understanding the Adult Women’s Recollections of Growing up in a Family Where There Was Domestic Violence

#### 3.1.1. Living with Contradictions, Double Binds and Dilemmas of Love and Abuse

Across all their stories, the women described how they experienced and lived the co-existence of two intense, paradoxical, and unresolved experiences—a contradiction organised around love and abuse with double binds and dilemmas. This experience of contradictions, double binds and dilemmas was labelled as a core category in line with the study’s grounded theory methodology. The woman questioned—how can my mother love my father and be so afraid of him? How can my mother protect me from my violent father and then insist that I love him and respect him? Others spoke about being violently assaulted by their father, having murderous rage against him, and yet understanding this beating as a way of their father wanting to bring them up well.

*“Because I used to see my mum... with all that fear and all that bitterness and then I used to see her so full of love for him... that I used to say... but did mum really love him or because she is afraid of him... Maybe she’s faking (the love) because she is afraid?”*

(Seana) [2] (p. 234).

*“and I used not to respect the curfew that he gave me and when I used to return home, I used to get butchered… literately, he used to beat me with the dog’s leash….beating me with his fist, in my eyes, in my stomach, everywhere…“During that time, I used to pray to Jesus so that he has an accident and he dies”…”and I had a good childhood...he was not violent… I was the apple of his eye…… I guess as I grew up, he wanted to raise me well”*

(Farrah) [2] (p. 2).

Grych and Fincham’s cognitive-contextual framework [54] can throw light on how children attempt to make sense of their terrifying, paradoxical experiences of love and abuse. At the same time, being so fearfully aroused, and because their relationships with their parents were so essential to them for their survival, they might have felt in a double bind [81,82], unable to meta-reflect on these dynamics until much later, when they became adults and had helped to process these experiences.

As some of the participants shared, “Trying to make sense came only much later, as an adult” (Mary). For these participants, their childhood was about surviving day by day, which many times was like trying to predict the unpredictable [83]. The process of understanding grew as they became older when they were likely to have the cognitive and emotional resources to support such understanding [17], when they were safe from the abuse and when they were in touch with different families. They came to the awareness that what they went through as children did not happen in all families. For some, psychological therapy was also helpful in processing what they lived through [84,85].

#### 3.1.2. Being Triangulated in the Parental Conflict

Another important context for these women’s experiences was being pulled into their parents’ conflict—what is referred to in the systemic literature as triangulation [15,52,53]. This concept is extensively referred to in the divorce and marital conflict literature and can be understood in the light of emotional security theory [44,45,50,51,86,87,88], but there is little reference to domestic violence in this literature. In this study, the women described how difficult it was not to take sides in their parents’ fights. It was almost impossible not to do so, even though it was painful to do so.

Some chose to get involved to protect their mother or their siblings:

*“My sisters didn’t do anything. But I was the one who always got involved between them. It is because these things bother you. It bothers me to see my mother getting hurt and beaten. So I used to go between them. I used to get punches… Even though I would be punched. As long as I managed to separate my mother away from him… That’s what I always wanted to do”*

(Marika) [2] (p. 213).

Others were physically and/or psychologically recruited by either parent. Geraldine recalled step-by-step how she and her brother were made to decide which of their parent should win the argument. Geraldine recalled that the process was very much like delivering a court judgement and that it was very hard for her to side with her mother as her father was a very good orator, like an extremely good lawyer, and by comparison, her mother was “exceedingly weak”. Moreover, Geraldine was terrified of her father and consistently took his side against her mother, focusing her energy on surviving and not getting beaten by him.

#### 3.1.3. Turning Points, Change and Resilience

With hindsight, the women also perceived their psychological growth and their resilience, together with all the terrible suffering and abuse that they experienced [89,90]. The women spoke about their sense of agency and competence in being in the middle of things [91,92]. Sometimes, when they got involved in their parents’ fights, they managed to protect their mother and stand up to their father. They experienced a growing sense of power and were validated by their family members’ approval (albeit sometimes implicit) for having stopped their violence. It was also around this time that some recalled that problems with their father began when they started rebelling and confronting him and standing up to the abuse as a way of fighting the helplessness of childhood [93,94].

Almost all the participants mentioned that growing older and understanding more of what was happening at home was a key turning point in their lives. As their social system widened to include friends, teachers and possibly mentors, they could make comparisons between their families and those of others, and they realised that there were other ways of being in a family [95,96]. Others, such as teachers, served as models on how they, as girls and women, could be different to their mothers:

However, understanding what was going on in their homes was not a straightforward process. Some of the participants spoke about their difficulties in naming their experiences as living through domestic violence unless they had help. Mary only realised that what she had gone through was domestic violence when she started working in a Domestic Violence Unit:

*“I started realising what this experience was all about when I started working in the domestic violence unit and I found myself working there because I had never wanted to work in the DV …, I wanted to work in child protection, with children …, I did not want to work in DV. And then when I found myself in the DV unit, it started dawning on me …, and we’re talking about a time when I had already got married and had the children and that I had done my degree and look how long it took me to see it as DV. And there I started learning …, there I started understanding …, when I used to read on DV, and how children experience DV, I started saying …, oh …, this is the same as I used to feel because I had never seen it in this way…… And I think, working in DV, reading and studying …, it was therapeutic for me because this was a way, I could understand and when you understand, you contain.”*

(Mary) [2] (p. 231).

These words underscore how naming and speaking aloud are important starting points for the processing of experiences, which are, until then, often nonconscious fragmented memories [97]. This also highlights how being informed by a trauma framework [67] might be helpful for practitioners working with children and adult survivors of childhood domestic violence. It was difficult for these participants to engage in a coherent meaning-making process without help from an adult or an adult sibling who was willing to have these conversations with them. Seeking help both as a child and as an adult was not easy. It was complicated by feeling afraid of their father’s reprisal and of being taken away by child protection social workers, feeling ashamed of not having a good family like other people had, and confused by apparent double-binds, contradictions and dilemmas as discussed above. Yet, participants also spoke about different processes of healing through attending formal psychotherapy, engaging in other therapeutic modalities, like meditation, engaging in sports and hobbies [98], and establishing meaningful relationships with mentors or significant others, like grandmothers, uncles, and aunts.

As the analysis of the data proceeded, it was apparent that understanding these women’s experiences was complex and thus, understanding these women’s relationships with their parents and siblings across time merited a description of the context of domestic violence given above. What follows is a more detailed description of their perceived relationships with their mothers, fathers, and siblings. Although each relationship is presented separately, each relational dyad needs to be understood in the context of all the other perceived relational dynamics across time.

### 3.2. Father-Daughter Relationship: Growing up in the Shadow of a Violent Father and Dealing with Dynamics of Fear, Love and Retaliation

What memories did the participants have of their fathers as children? The participants recalled memories of their father’s physical abuse towards their mother and frightening scenes of psychological abuse. For example, Sandra remembers herself as a 3-year-old trying to protect her mother from her father’s beatings when her father was almost going to kill her mother. Sandra remembered the continual fights between her parents, which did not end after their marital separation, which shows that ending the relationship does not end the domestic violence [99].

Jessica also remembered that her mother was afraid of her husband to the point that she needed her daughter to accompany her to the bathroom for fear that her husband would do something to harm her. Jessica remembered that her mother was having a lot of “accidents”, which she, as the daughter with the benefit of hindsight, understood as being a prelude to her mother’s murder. Jessica’s father murdered her mother when she was nine years old:

*“Yes, he was in the leisure business,… And he used to return home very late; he used to come home drunk and he used to come home to have sex with mummy; I don’t know, perhaps she did not let him or she used to be sleepy … At three o’ clock in the morning, it is not nice to wake up…, nowadays, I understand this …, and he started to beat her, and he started to beat her a lot and there were beatings in front of us, it was ugly …, Sometimes with a broom, sometimes on her head with the jewel box, throwing her here and there … so we saw a lot of this”*

(Jessica).

*“…, and she used to wake me up [at night] to go with her to the bathroom. That’s the extent that she was frightened [of him]…,”*

(Jessica) [2] (p. 136).

Claire did not remember any of her father’s physical abuse but clearly remembered incidents of coercion and control [100] with her father humiliating her mother. One incident which still fills her with anger today involved her father making her mother clean his spit spread all over the bathroom floor:

*“I remember once he had to go to the toilet to expectorate his catarrh and he spat everywhere, everywhere—maybe I was about 5 years—and then all of a sudden he told my mother—you can clean now—we will still go out. And I was still about 5 years—and I still look at him now and I feel anger inside me and I remember my mother cleaning after him …, he was …, he was cruel…”*

(Claire) [2] (p. 137).

Mary, on the other hand, spoke about having memories of a good relationship with her father when she was still a young child. Then their relationship got worse as she grew older, and started to make more sense of what was happening at home:

“… *I remember having a good relationship …, Around 3 years of age …, primary school age …, let’s say till 10 years of age; Yes, till around Holy communion (around 7yrs) …, I had a very good relationship with my father …, …I was always going around with him; I used to help him in his work, he used to take us out, he used to take us out for walks here and there …, he used to take us to his aunts and uncles, who for me, were old in years and elderly and they were interesting people and I used to stay exploring their house …, but then I think that the older that I got, the more I started understanding and seeing more what was happening in the house”*

(Mary) [2] (p. 143).

All these findings support other qualitative studies, which show how children, from a very young age, are keenly aware of what is going on, including all the different forms of violence [17,83,101]. In addition, these findings also concur with the handful of studies that not only report children’s negative perceptions of their fathers but also present a complicated mixture of both positive and negative and abusive practices from the father [25].

Negative experiences also included being sadistically and psychologically abused by the father, with the participants feeling absolute terror and fear, highlighting the significant overlap between child abuse and intimate partner violence [75,101,102]. Anita recalled one incident when her father hit her repeatedly with a garden hose, how painful and terrifying it was for her as a young girl:

*“fear, terror, we all used to be horribly scared of him …, he gave us one look and it was terrifying …, literally terrifying …, and it’s understandable since he would beat us terribly, that we will remember those beatings for the rest of our lives …, and really and truly for the rest of your life. Once …, he gave me this incredible beating with a hose …, I can still feel that beating to the present day …, it was one of those green garden hoses …, and you know why all this? Because my mum had left home …, so we were alone with him in the house …, he had sent me to buy something for him and I met my uncle, my mother’s brother and he gave me 10c and I being just a child, I bought some sweet sticks. They used to be 1c each so I bought 10 of them. I was going to divide them between us siblings …, maybe at that time we were still about four siblings …, I went back home and I was giving these sweets out …, and he told me “Where did you get the money from?” and I told him …, and let me tell you, he beat me with this rubber hose and each hit was a searing painful hit …, it was terrifying …,”*

(Anita) [2] (p. 138).

Such experiences were often accompanied by feelings of murderous rage towards their father of “*wishing that he were dead”* and then feeling guilty for wishing him dead. Others blamed themselves for their father’s beatings and his lack of appreciation [44,45]. Some others explained their fathers’ assaults as a way of his ensuring that they were brought up well—perhaps trying to make sense of cognitive dissonant experiences of horrific abuse by someone who is supposed to love you. Such experiences can also be understood in the light of Grych’s and Fincham’s cognitive-contextual framework, which focuses on how children try to understand and make sense of perceived threats [54].

What also came out strongly in the interviews was the participants’ yearning for their father’s love and support, highlighting the strength of the attachment bond and that the strength of the attachment bond does not predict the quality of the attachment relationships [46,47,48,49] “*Because at the end of the day, he is still your father… you cannot break the bond between you and him, even if he were a monster, you cannot*”. This quote is from Jessica, who, even though her father murdered her mother, did not want to lose him too and did not want to break the bond with him.

Such experiences illustrate how the participants were caught up with complex feelings—of love, fear, rage, and yearning for a connection with their father [17,25,103,104]. The daughters yearned for their fathers’ love, and they also might have felt pressured by their mothers to keep the relationship with their fathers as the mothers might have wanted to please their partners to prevent further abuse [105]. Daughters and mothers may have also been positioned by cultural and social beliefs around their responsibility for relationships and others’ emotional well-being [106,107].

### 3.3. The Mother-Daughter Relationship as a Child: Dynamics of Love, Abuse, Protection, and Betrayal

Most of the participants described having a loving relationship with their mother, which is also highlighted by studies such as Letourneau, Fedick, and Willms (2007) [108], where many mothers tried to make up for the violence that their children experienced through being more caring and more present for their children: “My mother is my world” (Seana) [2] (p. 169). For example, Donna knew that her mother would organise her and her siblings into a routine, like having an early bedtime, to protect them from hearing or experiencing further violence [105]. However, this did not actually protect them from witnessing the abuse that was perpetrated on the mother. Donna and Marika often stayed awake, trying to listen to the voices downstairs and sprang into defending their mother, often getting into the midst of the fights to protect her [109].

While this might have given them a sense of agency and a sense of power [110], it often left them feeling very distressed, worried about their safety, and wellbeing and that of their siblings.

Living in such circumstances, it is impossible to look at the children’s relationship with the mother without considering the domestic violence context. Despite feeling loved by the mother, the children felt that her protection and her love was limited. They felt that she did not protect them enough from the father’s violence and from taking on too much responsibility. They felt emotionally neglected [111]. The participants described their intense feelings of frustration and anger at their mother’s helplessness and her fear of standing up to the father’s abuse. They could not understand why she had not left, even as adults. This anger towards the mother was exacerbated when the daughters perceived the mother trying to minimise the father’s violent behaviour or when the mother encouraged the children to maintain some kind of contact with the father after separation. Few adult children were able to take a meta-position to the situation or take a more empathic position with the mother, understanding that perhaps their mother was protecting their father in order that they (the children) could form or maintain some kind of relationship with him or have a sense of family [105]. For many of these children, these complex feelings were very difficult to navigate without the help of a third other [112] with whom they felt safe enough to disclose what often was a family secret.

Some children felt betrayed by their mother in instances when she actively sided against them, for example, in Carmen’s case when her mother testified against her in court and in favour of her abusive stepfather. These findings are similar to the sexual abuse literature (e.g., Elliot and Carnes, 2001), which indicates that some mothers of incest and non-incest victims are not supportive of their daughters [113]. Other children spoke about their mother’s abusive behaviour, how, despite being a victim of the father’s violence, she also perpetrated physical and/or emotional violence on the daughters [114,115], highlighting to practitioners that both perpetrators and victims of domestic abuse could be abusive to their children. Anita, in fact, perceived her mother’s abuse and neglect as more damaging to her than her father’s severe physical abuse:

*“Let me tell you, more than picking on me …, let me tell you …, because my mother was worse and I feel more personally damaging than my father …, because her harassment was continuous …, it was continuous …, and I was a quiet child and introverted and she used to find me …, and I used to give her the opportunity to be her punching ball and whenever she felt like it she would lash out at me …, do you understand? …, it was continuously like that …, continuously …, Once my grandparents were going on a hike with my uncle who was coming for them with the car and my grandmother told me not to bother go back home to my mother. The next day my mother beat me with a shoe on my head until my head was bleeding, I had blood running down onto my school uniform and she sent me to school with my head still bleeding”*

(Anita) [2] (p. 151).

### 3.4. Sibling Relationships in Childhood: Protection, Abuse, Witnessing, Triangulation and Support

Amidst the desolation, fear and loneliness that these children experienced, some found their support and protection in their siblings [116]. To protect her siblings, Sara was the one to try and get back to the father in such a way that she and her siblings would have some kind of payback or redress:

*“I think that I am the only one of my siblings who managed to take money from him… I used to do it not as a form of stealing but to help the family (laughs)”*

(Sara) [2] (p. 155).

Hannah, too, spoke about the support that she had from her elder sister and how her elder sister always protected her. At the same time, witnessing her father physically abusing her sister was terrifying, and she experienced it as a form of silencing—that she would better comply and not challenge her father as otherwise, she would end up being beaten, too. Hannah, with hindsight, felt that there were times when she must have let her sister down, as she did not challenge her father as much as her sister did. This finding echoes the difference between siblings’ experiences—that although one might think that siblings living in the same family might have similar experiences, every child’s experience is unique [5,30]. Claire talked about feeling misunderstood by other siblings who did not have the same experiences of domestic violence as she had had:

*“Last time they [mother and sister] were talking to each other and they were saying how lively and happy my daughter is and my mum said—this is how Claire was- and my siblings didn’t want to believe her—who Claire? She’s so difficult and she does not smile a lot…. and then my mother didn’t say anything but she told them but this is how I know…”*

*“Yes, yes. First of all, I was the eldest and I have witnessed a lot of things that… till this very day, my siblings did not see and if the one after me saw them, she did not see the same things or she has not realised what she saw but—she either tried to forget them or in her own way, she’s trying to work it out in another way- all of us are doing it—the girls—three girls and a boy.”*

(Claire) [2] (p. 156).

Unfortunately, not all sibling relationships were supportive. The participants also spoke about their siblings being aggressive with each other [42]. Mary spoke about the rage that she felt that she and her brothers had:

*“What I remember most, was that in our house, there was a lot, a lot of anger, even between us siblings, between me and my brothers …, ferocious anger …, for example …, I don’t know …, we fight about something and then we used to go to each other’s rooms turn everything upside down and create havoc to each other’s things …, and that is the rage that we used to feel”*

(Mary) [2] (p. 155).

Similarly, Marika, one of the participants, nervously admitted that a while back, she too was beating her sister, and when she realised what she was doing and that she was behaving exactly like her father, she stopped herself.

### 3.5. The Daughter-Father Relationship in Adulthood: Cycle of Cut-Off and Connection, Dealing with Anger, Fear, Bitterness and Betrayal Together with Possibilities of Forgiveness and Redemption

As adults, almost all the participants still had some form of contact with their father, either because they were still living at home or because they still chose to keep some form of contact. This contact was experienced in a cycle that they described as a yearning for connection, making the connection but then deciding to cut off. In Donna’s case, this was often after her father would have verbally abused her and her mother during a visit that her mother would have pressured Donna to make to see her father. Donna talked about going back and forth between wanting to keep contact with her father and then realising that for her own good, she could not afford to have a relationship with him and be vulnerable again to his abusive behaviour:

*“I don’t know how to explain it…but in reality, it hurts…because you would wish that things were different…and you do wish that things one day will be different but then you rationalise and it does not…. It’s like you do recognise the need inside you but you know it is not helpful… I believe that it is that feeling that every girl feels about her father, that he should be in her life, but then reality kicks in and you realise that it cannot be possible…”*

(Donna) [2] (p. 168).

Similarly, Seana described herself as a yo-yo, shifting from here and there. She felt that she needed to remember the fear and terror that she felt during the last domestic violence incident so that she would not feel guilty about her decision not to speak to her father. However, a few days later, when her father contacted her and spoke to her in a civil manner, she felt that she had to give him a second chance because he was still her father. All these findings echo the earlier childhood experiences of hating the father and wishing him dead but then also wanting to maintain the bond with him. For those who still experienced some form of abuse from their father, the contradictions, double-binds and dilemmas of love and abuse persisted into adulthood. Such experiences highlight the complexities and challenges of adult child-parent relationships as domestic abuse continues to have a defining influence on the adult child’s relationships as well as on their well-being. All such experiences need to be taken into account by practitioners working with adult children living in domestic violence contexts.

With time, there were also relational experiences of transformation of forgiveness and reconciliation [117,118] between daughters and fathers, with them building a relationship where there was now mutual respect. This happened in the lives of three of the participants, Geraldine, Farrah and Sandra, where both the fathers and the daughters experienced a psychological change, especially the fathers who showed the daughters that they were taking responsibility for the harm that they had inflicted on their children [23,119]. The daughters also said that the rapprochement with the father was possible because they (the daughters) had processed some of their earlier adverse experiences with therapy or with mentors. It was also sometimes initiated by the daughter when there was illness or death of a family member and facilitated by the father’s respectful attitude [120].

*“…because when he was dying of cancer, I used to visit him every day- at the end I had grown very close to my father……after my husband got murdered, my father realised that he did wrong. He would tell me ‘you never wanted to get married, you married because of me…… He used to tell me ‘bring your daughter home and we will take care of her for you—go out, study—do whatever you need to do for yourself”*

(Farrah) [2] (p. 170).

Other participants, like Hannah, spoke about what seemed like her father’s redemptive process through grandparenting. Hannah spoke of the love that her father showered on her niece and how much the young child loved him. Although, at times, this saddened her as her father’s love was something that she would have wanted as a child, she was also happy for her father to have this relationship. To our knowledge, to date, this is an underexplored area in the domestic violence literature, and future research could explore in more depth the stories of men who have been abused and who have turned out to be good enough grandfathers. In contrast to the above, Hannah and her sister have given up on having a good relationship with him. Although she visits her parents regularly, she still finds them difficult to relate to:

*“yes. I can say that we don’t really get on well with each other. I can say we don’t know anything about each other; we don’t talk too much to each other. Ok I go home, and I have to live with them when I go home for a visit. But now I am an adult. So when I was a child, he could control me. Now he cannot and I don’t even care, because whatever he says, he thinks that he is always right. He is always perfect, anything and anybody else can be only wrong, and our relationship is still bad and this is not going to change because I have tried and my sister has tried so many times to make it better but it is not enough for us to try and change. It cannot be one-sided and my father is not going to change, so our relationship is just going to remain like that or worse, unfortunately”*

(Hannah) [2] (p. 163).

Of course, while some fathers and daughters could transform their relationship into a relationship with mutual respect, across time and with effort, other adult children maintained some kind of contact with their parent out of a moral obligation as daughters rather than because of an emotional attachment [121]. The distance from the violence at home gave them respite, but they still struggled with a complex array of strong emotions when getting in touch with their fathers and families again.

### 3.6. The Daughter-Mother Relationship in Adulthood: Persistence of Anger and Sadness, Love and Protection of the Mother and Witnessing the Mother-Daughter’s Relationship Transformation

*“The relationship with my mother is complicated”*—this is how most of the women described their current relationship with their mother. Although they loved their mother and described their relationship as good, they were still full of sadness at her limited protection, feeling resentful and carrying a sense of loss at being burdened with protecting her from the father’s violence [29]. So, while cognitively, Donna acknowledged the suffering that their mother went through, she still wished that her mother was more emotionally available to her. At the same time, despite these complications, Donna saw herself and her mother working out a new relationship between them. Donna was trying to re-position herself as a daughter in relation to her mother despite slipping back into a parental role again at times. Donna also appreciated the fact that her mother was keen to provide her with more emotional and material support.

On the other hand, even though Marika acknowledged her mother’s suffering, she was still very much preoccupied with anger against her mother and feeling let down by her. Perhaps she was still very much caught up with waiting for her mother to finally give her what she needed and needed, living what Fishbane refers to as the “spell of childhood” [122]. Sara also felt her anger at her mother intensify when her mother showed signs that she was willing to accept her husband back into the family home. Sara could not fathom how her mother could still love her father after the abuse that she (the mother) and the children suffered [123]. On the other side of the spectrum, Geraldine’s mother was still very much consumed by the anger towards her husband, and her daughter had a hard time having her mother accept or at least understand that it was important for the daughter to have a good relationship with both parents. Thus, as the women grew up, triangulation in their family of origin’s dynamics remained, for the most part, a challenging process, especially if the threat of violence raised its head again or if the aftermath of violence was still very much raw. The relationship with the mother is inextricably tied to the domestic violence context and the other relationships with the family members.

### 3.7. The Relationship with Siblings in Adulthood: The Persistence and Significance of Early Family of Origin Roles

The adult sibling relationships seemed to echo the relationships that the women had as children. Sara, Jessica and Donna still supported their siblings and their mother (in the case of Sara and Donna). Marika worried about her younger sister, who seemed to cope by withdrawing and keeping everything inside [124]. Claire noticed how one of her sisters, who was close to their mother, was unsure whether to marry or not, as she was worried about her mother’s mental and physical health because of the abuse that she endured from her husband. Claire’s sister’s doubts indicated that her sister continued to be triangulated in her parents’ relationship. When the first author asked Donna about the losses and gains in the sibling relationship, given her caring role, almost as a parent to her sisters [91], Donna talked about the loss of the fun element in the sibling relationship as she felt that she was not able to relax with her siblings. In some ways, it felt like she had no siblings. On the other hand, she felt close to her siblings because she knew that they understood what they all went through, even though they never really talked about the violence, as she was afraid that they would become emotionally distressed [17].

Seana was estranged from her brother and sister who lived with their father and who took the father’s side against the mother and herself and in some ways, this was a loss of the sibling relationships too. Seana wished that one day they would repair their relationship, but she did not know how that could come about as long as their parents kept fighting in spite of the marital separation.

Marika, Donna and Seana also referred to siblings abusing each other as young adults. Donna shared how her youngest sister was often physically abusive to her middle sister. Seana referred to being abused by her brother. These findings are supported by research which shows that sibling violence in the context of domestic violence is present and that such violence continues into adulthood, too, even after the parental violence has stopped [31].

It seems that it was hard for the siblings to change their relationships from their childhood roles, even when the violence stopped. It can be argued that changing behaviours, such as not getting triangulated in family interactions, both in the past and in the present, and not taking responsibilities that are overwhelming, is very difficult. For these women, there were long-standing challenges which they regularly had to revisit. All of this has important implications for clinicians working in the area.

## 4. Discussion

Given that findings were presented and discussed in the context of previous studies, we will now offer our reflections on the implications for therapeutic practice, discuss the limitations and strengths of this study and share our ideas for future research.

### 4.1. Implications for Therapeutic Practice

The findings arguably illuminate how, in working with adult children who have lived in families where there was and is domestic violence, therapists and clients need time and safety to develop an understanding of the impact of violence on themselves and their relationships. Even though the interviewed women were adults, they still struggled with early family-of-origin patterns of relating, and if the abuse was ongoing by the parent/s and the siblings, and even if it was less frequent than they were children, the participants were thrown into a survival loop that went against them developing a reflective, mentalising position that helped with regulating their affect [125,126]. This means that focusing on the current abuse and on issues of safety might need to take priority in the therapeutic work, highlighting again the complexity of working with this client group and how complicated it is for the clients to live through these experiences. In addition, it may be useful for practitioners to have an appreciation of trauma theory as a road map in their journey with clients where fear and terror hold such a prominent place in the participants’ narratives.

Therapists also need to be ready to listen and witness complex, contradictory emotions that might be hard to empathise with—especially hearing about the sadistic cruelty that the children had to experience and hearing about the children’s yearning for the love of a parent. One of the challenges of navigating these treacherous waters is not to jump in to save them or rescue them from their relationships with their parents and siblings. Suggesting cut-off under the cloak of setting boundaries or blaming the parents or the siblings can also be unhelpful [127]. At the same time, if the parents or siblings are a threat to the client’s safety, the therapist needs to prioritise this with the client.

The adult women’s reflections on their relationships with their mother and their expectation that she should have protected them highlight the complexities of mothering in domestic violence but also somewhat continue to underline how children grow up expecting their mothers to fulfil their needs, care for them and protect them [17]. Perhaps one can understand this as a construction of gender [24] or the children’s way of expecting reassurance and support, at least from the other parent, who is the non-offending parent. They may not understand that this is a very difficult, if not impossible, task for mothers in the domestic violence context despite the fact that some mothers do manage to be emotionally present for their children to a certain extent. In turn, in working with mothers and victims of domestic violence, therapists can support mothers to forgive themselves for not protecting their children. They might have done the best that they could, and the responsibility of the perpetrator for domestic violence and its impact on the mother-child relationship needs also to be recognised [24]. This also includes the responsibility of the mother if she, too, has abused the children in the context of being a victim of domestic violence.

Mother-child interventions are, to date, the treatment of choice for a successful psychosocial recovery of both parties [103]. But if the voices of the children or adult children are to be given centre stage, then practitioners need to create the space to unpack these complex mother-child relationships, particularly the experiences of the children’s maltreatment. It may be necessary to have individual sessions first before having conjoint sessions, if at all. In working with the mother, these individual sessions may offer the parent the opportunity to process some of the difficult experiences they went through as victims of domestic abuse, to talk about their abusive parenting and slowly be encouraged to take responsibility for their behaviour and for safety. Similarly, the children may be helped to cope with some of their overwhelming experiences without feeling that they have to minimise their suffering out of loyalty to their parents. This applies to interventions in childhood as well as support for adult survivors of childhood exposure to abuse.

Hurt, trauma and betrayal were also common experiences in the adult women’s recollections, but experiences of forgiveness and reconciliation, with respect to the father as the perpetrator, were also present and somewhat unexpected by the interviewer. The topic of forgiveness and repair within the context of domestic violence deserves a paper in its own right, but practitioners might find these papers useful resources [120,122,127,128]. As mentioned above, a central theme in the literature and in clinical work is that the perpetrators own responsibility for the harm caused to others. But when that is not possible, or when parents and siblings might not be available for repair, acceptance of one’s parents’ limitations but not the abuse might be a process that therapists and clients engage in if this is meaningful for the clients.

The findings also highlight that practitioners need to ask each sibling about their unique experiences in their family, which might be different from those of their brothers and sisters. Practitioners also need to ask about all forms of violence, including sibling violence and child-to-parent violence, especially as the children become teenagers or young adults. Practitioners also can inquire about experiences of warmth, care, and support between siblings if this is the case. The findings have shown that there is scope for doing therapeutic work with siblings, especially when these have been supportive in childhood and still are supportive of each other in adulthood. In contrast to the silence and secrecy associated with experiencing domestic violence, sibling therapy can help put their experiences in words and stories that can help them integrate their contradictory experiences [5].

### 4.2. Reflections on the Limitations and Strengths of the Study and Ideas for Future Research

Given the sensitivity of the topic, participants were recruited through health and social care professionals. This decision, which privileged ethics, limited the extent of available participants. The fact that they were all volunteers might have contributed to a sample bias. In having contact with professionals, they might have been more reflective and/or more resilient or more troubled than other participants who might not have been in contact with such professionals. The fact that they were recruited from a Maltese cultural context, which has a strong family orientation [59], might limit the theoretical applicability of the findings to other European societies. These characteristics may diminish the extent to which the results are applicable for other adult children with childhood experiences of domestic violence. However, it is hoped that the contextualised accounts of the participants and the extensive citations of their experiences in the form of quotes might help the reader in deciding what is similar and different from their context. This would then facilitate the readers’ decision on the applicability of the findings. Finally, retrospective accounts have often been criticised for their lack of accuracy in terms of memory recall. However, we concur with the position that subjective interpretations are key in negative psychological sequelae rather than the conflict itself [19].

Along the same lines, it would be interesting if future research took a multi-informant view of parenting from the perspective of the mothers, fathers, and adult children across time. This would shed light on what each family member thought was meaningful in their experiences and what possibly they may have wanted the other person to understand of their experiences. It would also be interesting to look into the process of healing intergenerational wounds from this multi-perspective and to explore what helps and hinders this process. Another interesting line of research could include grandparenting in the context of past domestic violence experiences.

## Figures and Tables

**Table 1:** Demographic Information of Participants.

Pseudonym	Age	Birth Order	Employment	Relationship Status	Children
Geraldine	35	1st of 3 siblings	Care worker in residential care	Separated	None
Rose	26	2nd of 2	Clerk	Single	None
Mary	37	1st of 4	Professional in caring profession	Married	2
Seana	27	2nd of 2	Sales executive	In a steady relationship	None
Sandra	30	1st of 3	Media executive	Single	None
Jessica	37	3rd of 4	Clerk	Separating and in a steady relationship	2
Anita	34	3rd of 5	Care worker	Married	2
Carmen	34	1st of 3 half siblings	Care worker	Married	2
Hannah	30	2nd of 2	Teacher	Married	None
Tori	21	Only child	Student	Single	None
Farrah	43	3rd of 3	Administrator	In a steady relationship	2
Marika	20	1st of 3	Clerk	Single	None
Sara	27	2nd of 4	Student in caring profession	Married	3
Claire	40	1st of 4	Teacher	Married	1
Donna	23	1st of 3	Student in caring profession	In a steady relationship	1

**Table 2:** Participants’ Profile re: the Violence They Were Exposed to and the Violence They Personally Experienced.

Pseudonym	Age	Exposed to Physical Assault: Father- Only on Mother; Mother-Only on Father or Mutually Violent Parents; Exposed to Emotional Violence	Experienced Physical and Emotional Abuse by Father, on Participant	Experienced Physical and/or Emotional Abuse by Mother, on Participant
Geraldine	35	Yes—often sadistic physical and emotional violence from father towards mother; emotional violence from mother to father	Severe physical, emotional and sexual abuse by father	Emotionally abused by mother
Rose	26	Yes, father-only physical assault and emotional violence	Physically abused by father; Retaliated against father with aggression during teens	No abuse indicated
Mary	37	Yes, father-only physical assault; mutually emotionally violent parents	Physically and emotionally abused by father	No abuse indicated
Seana	27	Father–only physical and emotional assault.	Physically and emotionally abused by father; Physical and emotional abuse was still ongoing as an adult; Physically and emotionally abused by siblings	No abuse indicated
Sandra	30	Father-only physical and emotional assault	Yes, physically abused by father; Retaliated with aggression during teens	Physical and emotionally abused by mother
Jessica	37	Father-only physical and emotional assault leading to the mother’s murder	Physically and emotionally abused by father; Emotional abuse by father was ongoing as an adult	No abuse indicated
Anita	34	Father-only physical and emotional assault; Mother emotionally violent	Physically and emotionally abused by father	Severe physical, emotional abuse and neglect by mother
Carmen	34	Step-father only physical and emotional assault	Severely physically and emotionally abused by step father	Severe physical, emotional abuse and neglect by mother
Hannah	30	Father only physical assault	Physically and emotionally abused by father	No abuse indicated
Tori	21	Witnessed father holding gun to mother; Mutual emotionally violent parents;	No abuse by father	Severe physical, emotional abuse by mother
Farrah	43	Father-only emotional assault on mother	Severe physical and emotional assault by father	No abuse indicated
Marika	21	Father-only physical and emotional assault	Severe physically and emotionally abused by father; Emotional abuse was ongoing; Was aggressive towards younger sibling	No abuse indicated
Sara	27	Father-only physical and emotional assault	Severely physically and emotionally abused by father	No abuse indicated
Claire	40	Father-only physical assault; parents mutually emotionally violent	Physically and emotionally abused by father	Physical abuse by mother but not considered as severe as that of father
Donna	23	Father-only physical and emotional assault Emotional assault on mother is ongoing when mother opts to meet him	Physically and emotionally abused by father	No abuse indicated

## Data Availability

More information about the data presented is available on request from the corresponding author. The data are not publicly available due to ethical reasons.
